# Psychotrophic Medication Prescriptions and Polypharmacy in Geriatric Patients Followed up in a Home-based Health Care Setting

**DOI:** 10.1192/j.eurpsy.2024.494

**Published:** 2024-08-27

**Authors:** S. Tanyeri Kayahan, A. Kayahan

**Affiliations:** Psychiatry, Yalvaç State Hospital, Turkish Republic Ministry of Health, Isparta, Türkiye

## Abstract

**Introduction:**

By 2050, one out of every six people in the world will be 65 years or older. Chronic diseases and associated multiple drug use are common in elderly. The use of five or more drugs is called polypharmacy and it’s reported between 40-90% in the elderly. The Beers Criteria is the American Geriatrics Association’s guide to current recommendations regarding the safety of pharmacotherapy in older age. Being a part of community-based health services in Türkiye since 2005, “Home-based Health Care Services” is a program in which patients, who are mostly elderly and have difficulty in accessing health institutions, access medical services at their homes.

**Objectives:**

In our study, it was aimed to examine the chronic disease diagnoses and prescriptions of patients aged 65 and over, registered in a home-based health care unit, in terms of psychotropic drugs and polypharmacy, and to evaluate the compliance of their psychiatric prescriptions with the Beers Criteria.

**Methods:**

Sociodemographic, psychiatric diagnosis and treatment prescription and home-based health service-specific data were collected from the electronic files of home-based health care unit patients. Chronic diseases were scored according to the Modified Charlson Comorbidity Index (mCCI). The last 6-month prescriptions obtained from the electronic patient files were scanned and included in the analysis. In statistical analysis using SPSS Version 25, a p-value of significance <.05 was determined.

**Results:**

As of February 2023, 229(83.2%) of 275 patients aged 65 and over constituted the research sample. The mean age of the sample, half of whom were considered as oldest-old(85 years and older), was 83±7.97(median=86,IQR=10.75), 69.9%(n=160) were women and 97.8%(n=224) were diagnosed with at least one chronic disease. The mean mCCI scores were 5.30±1.11(median=5.50, IQR=1.0). Polypharmacy was detected in 78.6% of the sample(n=180), among half(n=114) of whom at least one psychotropic was prescribed, drugs not recommended to be prescribed according to the Beers Criteria in elderly patients were 46%(n=52). Prescription rates were as follows: anti-dementia- 21.5%(n=49), antidepressants- 31.1%(n=71), antipsychotics- 21.5%(49) and benzodiazepines- 5.3%. Most frequently prescribed antidepressant was escitalopram 49.2%, while most frequently prescribed antipsychotic was quetiapine 29.4%. The frequency of quetiapine prescription increased significantly in patients with dementia (Χ²(1)=29.54, p<.001) and insomnia (Χ²(1)=13.11,p<.001).

**Conclusions:**

The frequency of polypharmacy was found to be closer to the higher values reported previously. Almost half of the sample had a prescription for psychotropic drugs, and one out of two of these prescriptions did not meet the Beers Criteria. Considering the aging population, it will be of great importance for clinicans to carefully evaluate psychotropic prescriptions and polypharmacy.

**Disclosure of Interest:**

None Declared

